# Spondylodiszitis und epiduraler Abszess

**DOI:** 10.1007/s00117-021-00814-6

**Published:** 2021-02-11

**Authors:** R. Rotzinger, R. Omidi, H. Gebhard, K. Shariat, F. Ahlhelm

**Affiliations:** 1 Abteilung Neuroradiologie, Zentrum für Bildgebung, Kantonsspital Baden AG, Im Ergel 1, 5404 Baden, Schweiz; 2 Departement Chirurgie, Kantonsspital Baden AG, Baden, Schweiz; 3 Klinik für Traumatologie, Universitätsspital Zürich, Zürich, Schweiz; 4 Klinik für Neurochirurgie, Kantonsspital Winterthur, Winterthur, Schweiz

**Keywords:** Wirbelsäule, Bandscheiben, Spondylitis, Magnetresonanztomographie, Spinale Entzündungen, Spine, Intervertebral disc, Spondylitis, Magnetic resonance imaging, Spinal cord inflammation

## Abstract

**Klinisches/methodisches Problem:**

Die Spondylodiszitis ist eine Entzündung der intervertebralen Bandscheibe, die beim Erwachsenen aufgrund der Vaskularisation in der Regel mit einer Spondylitis der angrenzenden Wirbelkörper einhergeht und klinisch häufig nur unspezifische Symptome wie Rücken- oder Nackenschmerzen zeigt. Sie kann erregerassoziiert von verschiedenen Pathogenen, v. a. Bakterien, verursacht werden. Dabei können ein oder mehrere Bewegungssegmente betroffen sein. Die Infektion kann epidurale Abszedierungen verursachen und sich auch auf umliegende Kompartimente ausbreiten. Die Radiologie, und hier insbesondere die Magnetresonanztomographie (MRT), spielt eine wichtige Rolle bei der Primärdiagnostik und im Rahmen von Verlaufsbeurteilungen zur Überprüfung des Therapieansprechens. Die Therapie beruht auf konservativen (Antibiotika) und invasiven Ansätzen, einschließlich einer Operation. Die interventionelle Punktion und Drainage ist v. a. in frühen Stadien der Abszessbildung eine vielversprechende Alternative.

**Radiologische Standardverfahren:**

Magnetresonanztomographie (MRT), Computertomographie (CT), nuklearmedizinische Verfahren, konventionelles Röntgen.

**Leistungsfähigkeit:**

Die MRT hat den höchsten Stellenwert. CT und nuklearmedizinische Verfahren können ergänzend und im Fall bestehender Kontraindikationen zur MRT eingesetzt werden.

**Schlussfolgerung:**

Bei adäquater Diagnostik und Therapie hat die Spondylodiszitis eine gute Prognose. Neben der gezielten bzw. kalkulierten medikamentösen Therapie steht beim epiduralen Abszess die invasive Therapie im Vordergrund. Die interventionell-radiologische Punktion und Drainage (auch zur Keimidentifikation für die gezielte Antibiotikatherapie) können frühzeitig eine schonende Alternative zur chirurgischen Sanierung darstellen.

## Abstract (en)

**Clinical/methodological problem:**

Spondylodiscitis is an inflammation of the intervertebral disc, which in adults is generally associated with spondylitis of the adjacent vertebrae. It often presents clinically with nonspecific symptoms such as back or neck pain. It may be caused by various pathogens, especially bacteria. One or more vertebral segments can be affected. The infection can spread to surrounding compartments and can lead to epidural abscesses. Radiology, in particular magnetic resonance imaging (MRI), plays an important role in the diagnostic work-up and in the follow-up to monitor response to therapy. Treatment consists of conservative (antibiotics) and invasive approaches, including surgery. Interventional puncture and drainage is a promising alternative to surgery, especially in early stages of abscess formation.

**Standard radiological methods:**

Magnetic resonance imaging (MRI), computed tomography (CT), nuclear medical procedures, conventional x‑ray.

**Performance:**

MRI has the highest value. CT and nuclear medical procedures can be used as a supplement to MRI and in patients with contraindications for MRI.

**Practical recommendations:**

With adequate diagnosis and therapy, spondylodiscitis has a good prognosis. In addition to targeted or calculated drug therapy, invasive treatment is the main focus, especially for epidural abscesses. Interventional radiological drainage can represent a less invasive alternative to surgical treatment.

## Anatomie

Die Wirbelsäule bildet die zentrale Achse des Körpers. Sie umhüllt das im Wirbelkanal liegende Rückenmark, welches sich über das Foramen magnum aus dem Hirnstamm bzw. der hinteren Schädelgrube samt den Hirnhäuten (Pachy- und Leptomeninx) fortsetzt und übergeht in die Kaudafasern bis ins Sakrum.

Erregerassoziierte spinale Entzündungen können entsprechend relativ schnell zu einer Beteiligung des zentralen Nervensystems und vorher seiner Hüllen führen.

Die Wirbelkörper sind dorsal über paarig angelegte obere und untere Facettengelenke verbunden. Diese formieren sich an den nach seitlich gerichteten oberen und unteren Gelenkfortsätzen. Zusammen mit den nach dorsal gerichteten Dornfortsätzen sind sie Ursprung und Ansatz der stabilisierenden autochthonen Rückenmuskulatur. Nach ventral sind die Gelenkfortsätze über den vorderen Wirbelbogen mit dem Wirbelkörper verbunden. Zudem verlaufen die Ligamenta flava als stabilisierende Bänder zwischen den Wirbelbögen und das vordere und hintere Längsband an der Vorder- und Rückseite des Wirbelkanals. Letzteres liegt dem Wirbelknochen nicht vollständig an und unterteilt den Epiduralraum zwischen Wirbelknochen und Dura mater in ein vorderes und hinteres Kompartiment. Die Arachnoidea ist innen relativ fest mit der Dura mater verwachsen, so dass ein Subduralraum physiologischerweise nicht existiert (Ausnahmen: iatrogen bei Myelographie oder beim subduralen Empyem). Zwischen Arachnoidea und Pia mater, die als dünne Haut oberflächlich dem Myelon direkt aufliegt, liegt der Subarachnoidalraum.

Die arterielle Versorgung der Hals- und Brustwirbel erfolgt aus Ästen der Arteria subclavia, die der Lendenwirbel, des Kreuz- und Steißbeins aus Ästen der Aorta abdominalis und der Arteriae iliacae internae. Die Endarterien, welche im Kindesalter die Bandscheiben versorgen, obliterieren in der Adoleszenz, so dass die Bandscheibe beim Erwachsenen keine direkte Blutversorgung besitzt. Die venöse Drainage erfolgt über einen inneren und äußeren Venenplexus, die über klappenlose, paravertebrale Venen (Batson-Venenplexus) mit tiefen Thorax‑, Bauch- und Beckenvenen verbunden sind.

## Epidemiologie

Die erregerassoziierte Spondylodiszitis ist mit einer Inzidenz von etwa 1:250.000 für etwa 3–5 % aller infektiösen Skeletterkrankungen verantwortlich. Sie ist die häufigste Manifestation der hämatogen gestreuten Osteomyelitis. Männer sind etwas häufiger betroffen als Frauen (Verhältnis etwa 1,5–2:1; [1]).

Die infektiöse Spondylodiszitis zeigt eine zweigipflige Altersverteilung. Die kindliche Spondylodiszitis tritt meist zwischen dem zweiten und achten Lebensjahr auf. Aufgrund der Blutversorgung der kindlichen Bandscheibe steht die hämatogene Keimbesiedelung im Vordergrund. Man kann bei Kindern eine isolierte Diszitis ohne begleitende Spondylitis beobachten [2]. Die Spondylodiszitis im Erwachsenenalter betrifft meist Patienten zwischen 50 und 70 Jahren, wobei alle Altersgruppen betroffen sein können. Im Erwachsenenalter spielt auch die Keimbesiedelung per continuitatem, z. B. nach chirurgischen Eingriffen an der Wirbelsäule, eine Rolle. Sie betrifft zwischen 25–30 % aller erregerassoziierten Spondylidiszitiden im Erwachsenenalter [1].

## Pathogenese

Der klassische Infektionsweg ist die hämatogene Keimbesiedelung [3]. Typischerweise kommt es beim Erwachsenen im Rahmen einer erregerassoziierten Spondylodiszitis zu einem Befall der Wirbelkörperendplatten und anschließend zu einer Ausbreitung auf die angrenzende Bandscheibe. Bei Kindern betrifft die Infektion aufgrund der Durchblutung hingegen meist zunächst die Bandscheibe.

**Figure 1:**
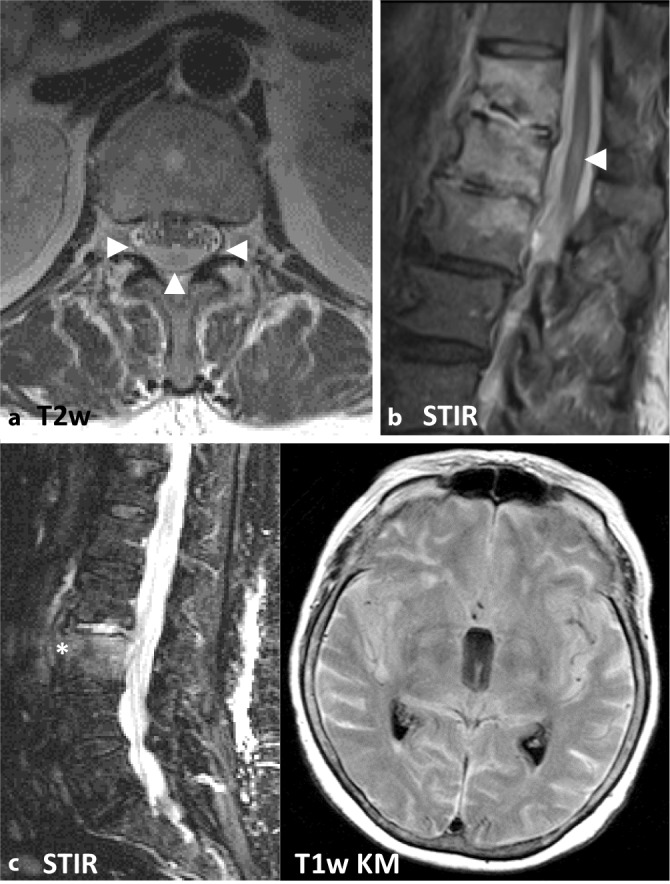

Unabhängig vom Alter kann es anschließend zu einer Ausbreitung des Infektionsgeschehens nach paravertebral, subdural und epidural kommen [2]. Hierbei sind als weitere Komplikationen, neben einer paravertebralen bzw. epiduralen Abszedierung, subdurale Abszesse sowie eine Ausbreitung in das zentrale Nervensystem möglich, beispielsweise in Form einer Meningitis, Myelitis oder Enzephalitis (Abb. 1; [4]).

Der Spondylodiszitis liegen in erster Linie bakterielle Erreger zugrunde, meist im Rahmen urogenitaler, dermaler oder respiratorischer Infekte. Die häufigsten Erreger betreffen *Staphylococcus aureus* (bis zu 60 %), gefolgt von Enterobakterien (bis zu 30 %), seltener z. B. *Staphylococcus epidermidis, Haemophilus influenza* oder Streptokokkenspezies [3]. Im Rahmen eines epiduralen Abszesses finden sich hauptsächlich *Staphylococcus aureus* (etwa 64 %) und Streptokokkenspezies (etwa 7 %; [5]).

Deutlich seltener spielen Mykobakterien, Pilze und Parasitosen eine Rolle, allen voran *Mycobacterium tuberculosis*. Pilzinfektionen können z. B. im Rahmen einer Candidose, Aspergillose, Coccidiomycose, Blastomycose, Cryptococcose oder Trichosporiose auftreten [6].

Infektionsfoki sind vorwiegend die lumbalen Segmente der Wirbelsäule (etwa 58 %), gefolgt von den thorakalen (etwa 30 %) und den zervikalen Abschnitten (etwa 11 %). Multisegmentale Manifestationen treten nur selten auf (in etwa 4 % der Fälle) und sind eher mit einem atypischen Erregerspektrum assoziiert [7].

Zervikale Verlaufsformen sind häufiger mit intravenösem Drogenabusus vergesellschaftet, thorakale Manifestation häufiger mit einer tuberkulösen Infektion. Die tuberkulöse Spondylodiszitis bzw. Spondylitis betrifft häufig mehr als zwei Segmente und kann ähnlich einer Pilzinfektion insbesondere die dorsalen Wirbelanteile betreffen, wie die hinteren Bogenanteile bzw. Wirbelfortsätze [1].

## Risikofaktoren

Zahlreiche Risikofaktoren werden mit dem Auftreten einer erregerassoziierten Spondylodiszitis in Verbindung gebracht. An erster Stelle ist allerdings Diabetes mellitus zu nennen. Ferner spielen intravenöser Drogenmissbrauch, katheterassoziierte Infektionen, chirurgische Eingriffe in der Vorgeschichte, infektiöse Endokarditiden, Harnwegsinfektionen und chronischer Alkoholismus eine Rolle. Erkrankungen, die mit einer Immunschwäche des Patienten einhergehen, spielen ebenfalls eine größere Rolle [8]. Für das Auftreten einer subduralen Abszedierung werden neben Diabetes mellitus insbesondere Leberzirrhose, chronisches Nierenversagen und infektiöse Endokarditiden diskutiert [5].

## Klinik

Die Symptome der Spondylodiszitis sind sehr unspezifisch. Rücken- oder Nackenschmerzen sind häufig, können jedoch in bis zu 15 % der Fälle fehlen. Typisch sind außerdem konstante Schmerzen, die sich nachts verschlimmern, differenzialdiagnostisch zum Osteoidosteom. Radikulär ausstrahlende Schmerzen sind nicht ungewöhnlich und können zu Fehldiagnosen sowie unnötigen Eingriffen führen. Fieber tritt nur bei etwa der Hälfte der Patienten auf. Neurologische Defizite sind in etwa einem Drittel der Fälle vorhanden und eher mit einer verzögerten Diagnosestellung, dem Auftreten epiduraler Abszedierungen, Manifestationen im Bereich der Halswirbelsäule oder einer tuberkulösen Spondylodiszitis bzw. Spondylitis assoziiert. Neurologische Symptome können von sensorischen Defiziten, über Radikulopathien, bis hin zur Querschnittsymptomatik bzw. zum *Konus-Kauda-Syndrom *mit resultierender Harn- und Stuhlinkontinenz reichen [9].

## Therapie

In der Behandlung der unkomplizierten, erregerassoziierten Spondylodiszitis steht die konservative Therapie im Vordergrund. Sie besteht aus antimikrobieller Pharmakotherapie und nichtpharmakologischer Behandlung, wie z. B. Physiotherapie. Aufgrund zunehmend komplexer Resistenzlagen im Erregerspektrum ist eine leitliniengerechte Antibiotikatherapie zwingend [8]. Dabei steht die gezielte Antibiotikatherapie mit Erregernachweis an erster Stelle. Dieser gelingt jedoch nur in 49–83 % der Fälle, bei einem chronischen Verlauf seltener als bei einem akuten. Eine wesentliche Ursache für einen ausbleibenden Erregernachweis stellt eine vorausgegangene systemische Antibiotikatherapie dar [10].

Die aktuelle antibiotische Therapieempfehlung der Deutschen Wirbelsäulengesellschaft (DWG) und der Deutschen Gesellschaft für Orthopädie und Orthopädische Chirurgie e. V. (DGOOC) bezieht sich auf die seit 2015 geltenden Empfehlungen zur Antibiotikatherapie der Infectious Diseases Society of America (IDSA; [11, 12]). Sie ist über mindestens 6 Wochen aufrechtzuerhalten. Bei tuberkulöser Infektion wird hingegen eine Therapie als Vierfachtherapie über 2 Monate mit Isoniazid, Rifampicin, Pyrazinamid und Ethambutol empfohlen, gefolgt von einer 7‑monatigen Therapie mit Isoniazid und Rifampicin [11]. Im Fall eines medikamentösen Therapieversagens, bei therapierefraktären Schmerzen, beginnenden neurologischen Defiziten oder bei spinaler Instabilität kann eine chirurgische Sanierung indiziert sein [8].

Eine Empfehlung über den Antibiotikaeinsatz bei typischem Erregerspektrum bietet Tab. 1**.**

**Table 1:** 

Erreger	Therapie der 1. Wahl	Alternative Therapie
Staphylokokken, oxacillinsensibel	Flucloxacillin 1,5–2 g i.v. (tid/qid)	Vancomycin i.v. 15–20 mg/kg (bid)^a^
	Cefazolin 1–2 g i.v.(tid)	Daptomycin 6–8 mg/kg i.v. (qd)
	Ceftriaxon 2 g i.v. (qd)	Linezolid 600 mg p.o./i.v. (bid)
		Levofloxacin p.o. 500–750 mg (qd) und Rifampin p.o. 600 mg/d oder Clindamycin i.v. 600–900 mg (tid)
Staphylokokken, oxacillinresistent	Vancomycin i.v. 15–20 mg/kg (bid)^a^	Daptomycin 6–8 mg/kg i.v. (qd)
		Linezolid 600 mg p.o./i.v. (bid)
		Levofloxacin p.o. 500–750 mg (qd) und Rifampin p.o. 600 mg/d
*Enterococcus spp.,* penicillinempfindlich	Penicillin G 20–24 mio. IE i.v. über 24 h kontinuierlich oder in 6 Einzeldosen	Vancomycin 15–20 mg/kg i.v. (bid)^a^
	Ampicillin 12 g i.v. über 24 h kontinuierlich oder in 6 Einzeldosen	Daptomycin 6 mg/kg i.v. (qd)
		Linezolid 600 mg p.o./i.v. (bid)
*Enterococcus spp.,* penicillinresistent	Vancomycin i.v. 15–20 mg/kg (bid)^a^	Daptomycin 6 mg/kg i.v. (qd) Linezolid 600 mg p.o./i.v. (bid)
Β‑hämolysierende Streptokokken	Penicillin G 20–24 mio IE i.v. über 24 h kontinuierlich oder in 6 Teildosen	Vancomycin 15–20 mg/kg i.v. (bid)^a^
	Ceftriaxon 2 g i.v. (qd)	
Enterobacteriaceae	Cefepim 2 g i.v. (bid)	Ciprofloxacin 500–750 mg p.o. (bid)
	Ertapenem 1 g i.v. (qd)	Ciprofloxacin 400 mg i.v. (bid)

^a^Kontrolle Serumspiegel [11, 12]

Epidurale Abszesse werden meist sofort einer chirurgischen Sanierung zugeführt. Bei Patienten ohne neurologische Symptomatik oder ohne spinale Instabilität kann jedoch, unter strenger klinischer Verlaufsbeobachtung, zunächst eine medikamentöse bzw. konservative Therapie versucht werden [5]. Zudem kann die radiologisch-interventionelle Abszessdrainage eine schonende Alternative zur chirurgischen Sanierung darstellen [8].

## Bildgebung

### Konventionelles Röntgen

Die konventionell-radiologische Aufnahme ist in der radiologischen Praxis häufig der erste diagnostische Schritt in der Abklärung unklarer Wirbelsäulenbeschwerden. Beim Vorliegen anamnestischer und klinischer Warnhinweise (sog. „red flags“) sollte gemäß der nationalen Versorgungs-Leitlinie „*Nicht-spezifischer Kreuzschmerz*“ (S3) zum Ausschluss spezifischer Ursachen, die einen dringenden Handlungsbedarf erfordern können, unmittelbar eine weiterführende Diagnostik mittels CT- oder MRT-Diagnostik erfolgen. Solche „red flags“ umfassen z. B. eine begleitende B‑Symptomatik (wie Fieber, Schüttelfrost, Appetitlosigkeit oder Müdigkeit), i.v.-Drogenabusus, Immunsuppression, konsumierende Grunderkrankungen, kürzlich zurückliegende Wirbelsäuleneingriffe oder starken nächtlichen Schmerz [13].

Hinsichtlich der Beurteilung einer Spondylodiszitis weist eine konventionell-radiologische Aufnahme eine nur geringe Spezifität auf (57 %) und ist insbesondere im frühen Verlauf häufig falsch-negativ [11]. Als Basisuntersuchung kann sie dennoch sinnvoll sein. Im konventionellen Röntgen können nach Eysel und Peters 4 Stadien unterschieden werden: Meist stellt sich zunächst eine Verengung oder Erweiterung des Bandscheibenfachs dar, infolge von Destruktion oder Ödembildung der Bandscheibe (Stadium 1). Anschließend kommt es im Verlauf zu Unschärfe und Unregelmäßigkeiten der Endplatten im Rahmen fortschreitender Osteodestruktionen (Stadium 2). Fehlstellungen, mit Ausbildung einer segmentalen Kyphose (Stadium 3) bzw. einer Ankylosierung in einer mehr oder weniger ausgeprägten Kyphose (Stadium 4), können auf ein Fortschreiten der Erkrankung hinweisen. Ein erweiterter Weichteilschatten paravertebral kann Anhalt geben für eine beginnende oder erfolgte Abszedierung [4].

### Computertomographie (CT)

Die CT ist der konventionell-radiologischen Aufnahme in der Diagnostik der Spondylodiszitis überlegen, aufgrund höherer räumlicher und Weichteilkontrastauflösung. Neben der Beurteilung der knöchernen Zerstörung der Endplatten kann die CT eine beginnende oder erfolgte ossäre Sequesterbildung nachweisen. Außerdem kann die CT Hinweise geben auf eine entzündliche Reaktion der umliegenden Weichteilstrukturen, z. B. anhand von Flüssigkeitseinlagerungen in das paravertebrale Fettgewebe oder eine zunehmende Hypodensität der Bandscheibe [4]. Generell lassen sich entzündliche Veränderungen in der CT aber ebenso wie paravertebrale oder epidurale Abszesse besser nach intravenöser Kontrastmittelgabe diagnostizieren, wobei die CT der MRT bei der Weichteildiagnostik unterlegen ist. Eine Rolle spielt die CT insbesondere bei Kontraindikationen zur Durchführung einer MRT, bei bildgestützten Biopsien und als präoperative Bildgebung vor chirurgischer Sanierung [11].

### Magnetresonanztomographie (MRT)

Einen Überblick über ein mögliches MRT-Protokoll und typische Befunde gibt Tab. 2.

**Table 2:** 

Sequenz	Schichtdicke	Orientierung
T1w, T2w	3 mm (HWS), 4 mm (BWS, LWS)	Sagittal
T1w, T2w (3D)	≤1 mm (isometrisch)	Transversal^a^
STIR	3 mm (HWS), 4 mm (BWS, LWS)	Sagittal oder koronar
FS T1w nach KM i.v.	3 mm (HWS), 4 mm (BWS, LWS)	Sagittal und koronar; transversal^a^

*STIR* Short-Tau-Inversion-Recovery, *FS* fettgesättigt. *KM* Kontrastmittel

^a^Befundbezogen

**Figure 2:**
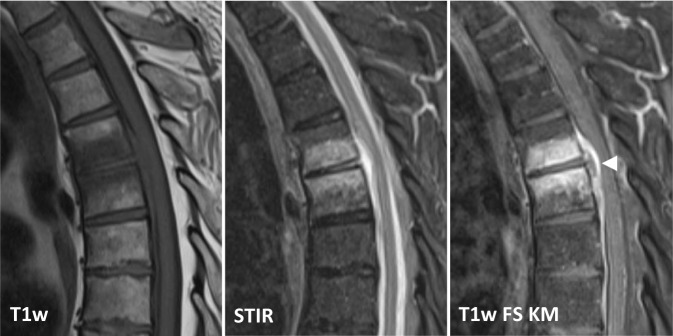

Die MRT ist mit einer Sensitivität von 96 %, einer Spezifität von 92 % und einer Genauigkeit von 94 % die Bildgebung der Wahl [4]. Noch vor knöchernen Destruktionen zeigt sich eine Zunahme des Flüssigkeitssignals der betroffenen Wirbelkörper und Bandscheiben im Sinne eines T1w-hypointensen, T2w-hyperintensen Ödems sowie in den T1-gewichteten Sequenzen eine Anreicherung von i.v.-Kontrastmittel (Abb. 2). Der Verlust des sog. „nuclear cleft sign“, einer T2w-Hypointensität der zentralen Bandscheibe, die von einigen als altersabhängig physiologischer Prozess, von anderen als erster Grad der Degeneration der Bandscheibe gewertet wird, kann mit einer Spondylodiszitis einhergehen, ist für eine solche jedoch nicht spezifisch [14].

Die Entzündungsreaktion lässt sich meist zunächst anterolateral im Wirbelkörper in der Nähe der Endplatten abgrenzen und zeigt nach Kontrastmittelgabe in T1-gewichteten Sequenzen eine teilweise inhomogene Kontrastmittel-Mehranreicherung. Das assoziierte Ödem betrifft hingegen typischerweise den größten Teil des Wirbelkörpers und der angrenzenden Bandscheibe [4]. Bei der tuberkulösen Spondylodiszitis bzw. Spondylitis kann die Bandscheibe weitgehend erhalten sein, da diese häufig vorrangig den Knochen, insbesondere die dorsalen Wirbelanteile, betrifft [15].

Einen Überblick über typische Befunde der pilzassoziierten Spondylodiszitis gibt Tab. 3.

**Table 3:** 

Anatomie	Korrelat in der Magnetresonanztomographie (MRT)
Wirbelkörper	Destruktion der Endplatten; T1w-Hypointensitäten, T2w-Hyperintensitäten, Anreicherung von i.v. Kontrastmittel; Osteolysen, Destruktionen, Erosionen
Paraspinalraum/Epiduralraum	Kleine paraspinale Abszessformationen; unscharf abgrenzbare Entzündungsreaktionen
Anteriore, subligamentäre Ausbreitung	Häufig
Beteiligung angrenzender Wirbelsegmente	Selten
Beteiligung mehrsegmentaler Wirbelsegmente	Häufig bei Coccidioidomykose
Deformitäten der Wirbelsäule (Gibbus)	Hauptsächlich bei Blastomykose

### Nuklearmedizin

Die nuklearmedizinische Abklärung spielt eine Rolle bei Kontraindikationen gegen eine MRT und bei milden Infektionsverläufen, bei denen in der MRT keine eindeutigen entzündlichen Veränderungen abzugrenzen sind. Die Tc-99m-Knochenszintigraphie bietet eine hohe Sensitivität (um 90 %), allerdings bei eingeschränkter Spezifität und räumlicher Auflösung [16]. Die Ga-67 Einzelphotonen-Emissions-Computertomographie (SPECT) kann insbesondere in Kombination mit einer konventionellen CT Ergebnisse liefern, die in Sensitivität und Spezifität einer MRT vergleichbar sind. Ähnliches gilt für die F‑18-Fluordeoxyglukose-Positronen-Emissions-Tomographie (FDG-PET; [4]).

## Differenzialdiagnosen

### Modic-Typ-1-Degeneration

Degenerative Prozesse der Bandscheiben sind häufig vergesellschaftet mit erosiven Veränderungen der angrenzenden Wirbelkörperendplatten. In der MRT zeigen Modic-Typ-1-Veränderungen ein endplattenassoziertes Knochenmarködem, was eine erregerassoziierte Spondylitis vortäuschen kann. Die Bandscheibe zeigt hingegen, im Gegensatz zur erregerassoziierten Spondylodiszitis, typischerweise keine T2w-Hyperintensität. Nach Kontrastmittelgabe i.v. können sowohl degenerativ entzündliche als auch erregerassoziierte Prozesse eine KM-Anreicherung der Endplatten zeigen. In diffusionsgewichteten MRT-Sequenzen stellt sich hingegen ein degenerativ entzündlicher Prozess eher durch eine lineare Diffusionsstörung an der Grenze zwischen hypervaskularisiertem und normalem Knochenmark dar, wohingegen bei einer erregerassoziierten Spondylodiszitis eher eine diffuse Diffusionsstörung der gesamten Ödemzone abzugrenzen ist [17, 18].

### Diskusherniationen

Eine akute intravertebrale bzw. intraspongiöse Diskusherniation (sog. *Schmorl-Knoten*) kann je nach Ausdehnung und Muster der Vaskularisierung und Entzündungsreaktion in der MRT schwer von einer erregerassoziierten Spondylodiszitis zu differenzieren sein. Für einen akuten Schmorl-Knoten spricht ein den Knoten konzentrisch umgebender, T2w-hyperintenser Ring. Die T2w-Signalalterationen betreffen typischerweise nicht die gesamte Bandscheibe. Knochendefekte, die auf eine der beiden Endplatten beschränkt sind, sprechen ebenfalls eher für das Vorliegen von Schmorl-Knoten [18].

Bandscheibenherniationen, bei denen sich Bandscheibengewebe vom Diskus loslöst und migriert (sog. *Sequester*), können eine Abszedierung vortäuschen. Neben spinalen Sequestern, die sich ähnlich einer epiduralen Abszedierung darstellen können, sind auch paravertebrale Sequester beschrieben, welche z. B. auf Höhe der Lendenwirbelsäule ein- oder beidseitige Psoas-Abszesse suggerieren können. Die Differenzierung in der MRT kann aufgrund der ähnlichen Signalgebung schwierig sein, wobei diffusionsgewichtete Sequenzen in der Regel helfen, die Befunde zu differenzieren [19].

### Wirbelkörperfrakturen

**Figure 3:**
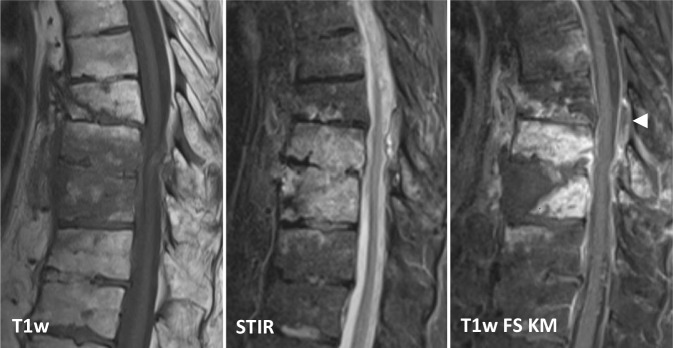

Frakturen können neben dem knöchernen Wirbel auch die Bandscheibe betreffen und mit einem begleitenden Hämatom paravertebral oder spinal einhergehen. Das Hämatom kann dabei ebenso wie eine paravertebrale Abszedierung Gaseinschlüsse enthalten. Befunde können in der CT-Diagnostik aufgrund von zentraler Verflüssigung, kapselartigem Granulationsgewebes und perifokaler Weichteilödematisierung einer Abszessformation ähneln (Abb. 3).

In der MRT lässt sich ein in der Regel ein T1w‑/T2w-hyperintenses Hämatom (*Cave:* Signalabhängigkeit von der Degradation) jedoch meist gut von einem T1w-hypointensen, T2w-hyperintensen Abszess unterscheiden [20].

### Neuropathische Arthropathie

Die neuropathische Arthropathie der Wirbelsäule (sog. *Charcot-Wirbelsäule*) ist ein destruktiver Prozess als Reaktion auf wiederholte Traumata, der bei Patienten mit verminderter Schmerzempfindung auftritt (z. B. bei *Diabetes mellitus, Syphilis* oder *Syringomyelie*). Am häufigsten sind die untere Brust- und die Lendenwirbelsäule betroffen, wobei mehrere Wirbelsegmente betroffen sein können. Klinik und Bildbefund können einer erregerassoziierten Spondylodiszitis ähneln. Im späten Stadium der Pseudarthrose kann es zudem zu paraspinalen Weichteilformationen kommen, welche eine paravertebrale Abszedierung suggerieren können. In der MRT weist das Bandscheibenfach und das umgebende Knochenmark in den T2-gewichteten Sequenzen in der Regel eine geringere Signalintensität auf als es bei der erregerassoziierten Spondylodiszitis der Fall ist. Zudem können Begleitbefunde wie Gaseinschlüsse im Bereich der Bandscheibe, eine assoziierte Spondylolisthese oder Facettenbeteiligung auf eine neuropathische Wirbelsäule hinweisen [18, 21].

### SAPHO-Syndrom

Das SAPHO-Syndrom beschreibt eine Kombination aus Synovitis, Akne, Pustulose, Hyperostose und Osteitis. Knöcherne Veränderungen finden sich vorzugsweise in der vorderen Brustwand (70–90 %) und der Wirbelsäule (etwa ein Drittel der Patienten). Zu typischen Befunden in der MRT gehören ähnlich der erregerassoziierten Spondylodiszitis fokale oder diffuse Signalinhomogenitäten des Knochenmarks, Unregelmäßigkeiten der Endplatten, eine Verengung des Bandscheibenraums, T2w-Signalhyperdensitäten bzw. eine Anreicherung der Bandscheibe nach i.v.-Kontrastmittelgabe. Weichteilschwellungen paravertebral können im Rahmen eines SAPHO-Syndroms auftreten, eine Abszedierung oder epidurale Beteiligung spricht hingegen gegen ein SAPHO-Syndrom. Klassisch für das Vorliegen eines SAPHO-Syndroms sind Erosionen der vorderen Wirbelwinkel oder Wirbelwinkelerosion in einem weiteren Wirbelsäulensegment [18, 22].

### Septische Arthritis

Die septische Arthritis stellt ein hochakutes Krankheitsbild und eine Notfallsituation mit hoher Letalität dar (11–50 %). Sie entspricht einer pyogenen Entzündungsreaktion des Gelenks, häufig im Bereich der kleinen Wirbelgelenke. Der am häufigsten nachgewiesene Erreger ist *Staphylococcus aureus,* gefolgt von Streptokokken. Nur bei 10–20 % der Fälle sind mehrere Gelenke betroffen. Bildmorphologisch ist die septische Arthritis häufig nicht von aktivierten degenerativen Gelenkveränderungen zu differenzieren [23].

Ähnlich der erregerassoziierten Spondylodiszitis kann es bei der septischen Arthritis zur Ausbreitung der Infektion in die umliegenden Kompartimente kommen. Dabei sind insbesondere paravertebrale und epidurale Abszesse typisch. Beim Nachweis einer epiduralen Abszedierung ist im Fall einer fehlenden Entzündungsreaktion in den Endplatten bzw. der Bandscheibe daher die septische Arthritis eine eher seltene, aufgrund der hohen Letalität aber wichtige, Differenzialdiagnose [23, 24].

### Non-Hodgkin-Lymphom

Das *Non-Hodgkin-Lymphom* findet sich im Bereich der Lendenwirbelsäule in 6–34 % im Rahmen einer systemischen Erkrankung. Primäre Lymphome der Wirbelsäule sind die Ausnahme. Eine ossäre Beteiligung findet sich in 7–25 %. Die Ausbreitung erfolgt durch direkte Expansion oder hämatogen, wobei der Batson-Venenplexus okkulten Lymphomen der Beckenlymphknoten die Möglichkeit des Befalls der Wirbelsäule ermöglicht, bevor sie klinisch selbst manifest werden [25].

In der MRT lässt sich häufig eine schlecht abgegrenzte, T1w-hypointense, T2w-inhomogene Läsion mit schrittweiser Ausdehnung und Durchbruch durch die Kortikalis beobachten. Das Lymphom kann sich so auf die paravertebralen und intraspinalen Kompartimente ausbreiten und Kalzifikationen zeigen [25].

## Fazit für die Praxis

- Die Spondylodiszitis gehört zu den wenigen spinalen Notfällen, die eine dringliche radiologische Abklärung erforderlich machen.
- Bei Kindern kann die Spondylodiszitis isoliert auftreten. Beim Erwachsen handelt es sich bei der typischen Form i. d. R. um ein Mischbild aus Spondylodiszitis und Spondylitis.
- Die erregerassoziierte Spondylodiszitis kann durch eine Vielzahl an Pathogenen hervorgerufen werden, von denen einige mit typischen Bildbefunden assoziiert sind.
- Neben der paraspinalen Beteiligung können auch intraspinale epidurale Abszedierungen sowie Senkungsabszesse beobachtet werden.
- Die frühzeitige Diagnose und medikamentöse Behandlung können dazu beitragen, eine chirurgische Sanierung und Komplikationen, wie Abszedierungen, Meningitiden, Empyeme, bis hin zu Enzephalitiden, zu vermeiden.
